# Supplementary material for: Visualizing the transiently populated closed-state of human HSP90 ATP binding domain
Source: Nat Commun. 2022 Dec 9;13:7601. doi: 10.1038/s41467-022-35399-8 (PMC9734131; doi:10.1038/s41467-022-35399-8)
Supplement: Supplementary file 1 — Supplementary Information [file 41467_2022_35399_MOESM1_ESM.pdf]

Visualizing the Transiently Populated Closed-State of Human HSP90 ATP Binding Domain

Supplementary Information

Faustine Henot[1,#], Elisa Rioual[1,2,#], Adrien Favier[1], Pavel Macek[1,3], Elodie Crublet[3], Pierre Josso[2], Bernhard Brutscher[1], Matthias Frech[4], Pierre Gans[1], Claire Loison[2,*], Jerome Boisbouvier[1,*]

Affiliation:
1. Univ. Grenoble Alpes, CNRS, CEA, Institut de Biologie Structurale (IBS), 71, avenue des martyrs, F-38044 Grenoble, France.
2. Institut Lumière Matière, University of Lyon, Université Claude Bernard Lyon 1, CNRS, F-69622, Villeurbanne, France.
3. NMR-Bio, 5 place Robert Schuman, F-38025 Grenoble, France.
4. Discovery Technologies, Merck KGaA, Frankfurter Straße 250, 64293 Darmstadt, Germany.
# These authors contributed equally to this work
* correspondence to be addressed to: claire.loison@univ-lyon1.fr and jerome.boisbouvier@ibs.fr

Table of Contents

Supplementary Table 1: Summary of structural restraints. p. 3

Supplementary Table 2: Definition of NOE-derived distance restraints specific of a single state. p. 4

Supplementary Table 3: Summary of NMR refined structure statistics. p. 6

Supplementary Table 4: Pairwise RMSD comparison of MD and X-ray representative structures. p. 7

Supplementary Table 5: Identification of characteristic distances of dimeric forms of HSP90α-NTD. p. 8

Supplementary Table 6: NMR-based structural calculations Supplementary the absence of helix-5 in ATP-lid closed state. p. 9

Supplementary Figure 1: Assigned 2D NMR spectra of HSP90α-NTD. p. 10

Supplementary Figure 2: HSP90α-NTD assignment. p. 11

Supplementary Figure 3: Summary of NOEs distance restraints. p. 12

Supplementary Figure 4: Assigned 2D ^{1}H-^{13}C SOFAST methyl TROSY of HSP90α-NTD mutants p. 13

Supplementary Figure 5: Intensity variation of characteristic NOEs. p. 14

Supplementary Figure 6: Root mean square fluctuations analysis of MD trajectories p. 15

Supplementary Figure 7. Time evolution of the violations of the NOE-derived distance restraints specific of either the open or the closed conformation for the 20 non-restrained molecular dynamics simulations starting from ATP-lid closed state conformers. p. 16

Supplementary Figure 8: Analysis of HSP90-NTD dynamics using relaxation dispersion experiments. p. 18

Supplementary Figure 9: Comparison of Energy landscapes of WT-, R60A-, R46A-HSP90α-NTD. p. 19

Supplementary Figure 10: Intensity variation of closed state characteristic NOEs in presence of ligands. p. 20

Supplementary Figure 11: Probability of helical structures along the sequence of the ATP lid, in 4 MD simulations depicting a transition of the ATP-lid from a closed to an open state. p. 21

Supplementary Figure 12: SEC-MALS analysis of uncleaved R46A- and WT-HSP90α-NTD p. 22

Supplementary Table 1: Summary of structural restraints used for structure calculation of WT-, R60A- and R46A-HSP90α-NTD.

		WT[(a)]	R60A	R46A
**Experimental distance restraints**				
**CH_3-CH_3**		**111**	**114**	**81[(b)]**
Sequential / local (≤i+4) / long range / ambiguous		4 / 12 / 75 /20	5 / 13 / 78 / 18	4 / 15 / 57 /5
**H_N-H_N**		**41**	**41**	**41**
Sequential / local (≤i+4) / long range / ambiguous		18 / 19 / 3 /1	18 / 19 / 3 / 1	18 / 19 / 3 / 1
**H-bonds**		**0**	**2**	**2**
**Experimental angular restraints**				
**Dihedrals**	φ, ψ	**36[(c)]+54[(a)]**	**54**	**36[(c)]**
**Restraints for rigid body**				
Dihedrals	φ, ψ	475 + 477[(a)]	477	475
Distances	C_α-C_α	13434 + 13434[(a)]	13434	13434

(a) Structure calculations using NMR restraints derived from WT-HSP90-NTD sample were performed using a two-state structure calculation protocol enabling each experimental distance restraint to be satisfied in either ATP-lid open- or closed-state or both states simultaneously[54]. The angular restraints, and distance restraints for the rigid body core (segments 11-97 and 137-223) were applied on both states simultaneously. Dihedral restraints, corresponding to helix-5 in the ATP-lid open state (from residues 128 to 136), were not applied for the closed ATP-lid state (See note (c) for further information).
(b) A total of 103 inter-methyl distance restraints were experimentally extracted using R46A-HSP90α-NTD sample to characterize the ATP-lid segment (from residue M98 to V136). Removal of R46/S129 hydrogen bond by mutagenesis leads to an increase of the population of the closed state, but NOEs corresponding to an ATP-lid in both open and closed conformations were detected simultaneously. Therefore, we used the previously refined solution structure with the ATP-lid in an open position (obtained using R60A-HSP90α-NTD sample) to identify and exclude 22 NOEs specifically assigned to the ATP-lid in an open state (see Supplementary Table 2). The remaining 81 NOEs were completed with backbone restraints previously obtained on WT-HSP90α-NTD sample for the final structure calculation using a single state structure calculation protocol.
(c) Structure calculation allowed us to identify large violations due to incompatibilities between helix-5 dihedral restraints and NOEs specific of the ATP-lid closed state (Supplementary Table 6). Therefore, the angular restraints from residues 128 to 136 were omitted for the final CYANA structure calculation of ATP-lid closed state using restraints derived from the R46A-HSP90α-NTD sample.

Supplementary Table 2: Definition of NOE-derived distance restraints specific of a single state.

A) NOE-derived distance restraints specific of the ATP-lid closed state. NOEs observed only for the WT- and R46A-HSP90α-NTD mutants and not for R60A-HSP90α-NTD sample. In the structure ensembles calculated with a two-state protocol using distance restraints derived from NOEs detected on WT- and R46A-HSP90-NTD, those restraints are satisfied only in the ATP-lid closed state and violated by more than 5 Å in the ATP-lid open state.

Restraint	Residue i	Pseudoatom i	Residue j	Pseudoatom j	d_{ij}^{max} (Å)[a]	d_{ij}^{viol} (Å)[b]
**1**	LEU64	HD2*	MET130	HE*	6.5	10.08
**2**	THR65	HG2*	ALA121	HB*	6.5	10.43
**3**	THR65	HG2*	ALA124	HB*	6.5	9.85
**4**	THR65	HG2*	ALA126	HB*	6.5	10.19
**5**	THR65	HG2*	MET130	HE*	6.5	10.19

B) NOE-derived distance restraints specific of the ATP-lid open state. NOEs observed for WT-, R46A-, or R60A-HSP90α-NTD samples. In the structure ensembles calculated with a two-state protocol using distance restraints derived from NOEs detected on WT- and R46A-HSP90-NTD, those distance restraints are satisfied only in the ATP-lid open state and violated by more than 5 Å in the ATP-lid closed state.

Restraint	Residue i	Pseudoatom Pi	Residue j	Pseudoatom Pj	d_{ij}^{max} (Å)[a]	d_{ij}^{viol} (Å)[b]
1	ALA24	HB*	MET119	HE*	8	11.87
2	ILE26	HD1*	THR115	HG2*	8	5.54
3	ILE26	HD1*	MET119	HE*	8	8.59
4	ILE26[c]	HD1*	ILE131[c]	HD1*	8	[f]
5	LEU29	HD2*	THR115	HG2*	8	6.63
6	LEU29	HD2*	MET119	HE*	8	6.16
7	LEU29	HD2*	LEU122	HD2*	8	8.89
8	LEU32	HD2*	MET119	HE*	8	4.52
9	LEU32	HD2*	LEU122	HD2*	8	6.24
10	LEU32[c]	HD2*	MET130[c]	HE*	8	[f]
11	LEU32	HD2*	ILE131	HD1*	8	9.65
12	THR36	HG2*	LEU122	HD2*	8	8.53
13	THR36	HG2*	ILE131	HD1*	8	11.65
14	ILE43	HD1*	LEU122	HD2*	8	9.42
15	ILE43[d]	HD1*	MET130[d]	HE*	8	[f]
16	ALA111	HB*	MET119	HE*	8	10.55
17	THR115	HG2*	VAL136	HG2*	8	6.64
18	MET119	HE*	VAL136	HG2*	8	8.76
19	LEU122	HD2*	VAL136	HG2*	8	8.31

Restraint	Residue i	Pseudoatom Pi	Residue j	Pseudoatom Pj	d_{ij}^{max} (Å)[a]	d_{ij}^{viol} (Å)[b]
20	LEU122	HD2*	LEU143	HD2*	8	7.98
21	LEU32	HD2*	THR115	HG2*	8	10.72
22	LEU32	HD2*	ALA126	HB*	8	8.53
23	MET119[e]	HE*	ILE131[e]	HD1*	8	[f]
24	LEU122[e]	HD2*	ILE131[e]	HD1*	8	[f]

[a] For each NOE cross-peak observed associated to a pair of residue {i,j}, d_{ij}^{max} defines the upper limit for the distance restraints applied for structure calculations.
[b] In the MD simulations, the violation relative to a given NOE-derived distance restraint differs from zero if the distance between the pseudoatoms Pi and Pj is larger than dij^{viol} (see more details Section 1.2.2 of Methods).
[c] NOEs not observed in the construct HSP90-R46A
[d] NOEs not observed in the construct HSP90-R60A
[e] NOEs not observed in the construct HSP90-WT
[f] Distance not used to calculate violation in the analysis of the MD trajectories.

Supplementary Table 3: Summary of NMR refined structure statistics for HSP90-NTD in ATP-lid open and closed states.

	Closed state (R46A-HSP90-NTD)	Open state (R60A-HSP90-NTD)
**RMSD to mean structure**[a]		
Backbone atoms (Å)	1.50 ± 0.26	0.83 ± 0.17
Heavy atoms (Å)	2.04 ± 0.26	1.27 ± 0.19
**Distance restraints violations**		
Average number of violations (>0.4Å)	0	0
Highest violation (Å)	0.18	0.26
**Ramachandran analysis**[b]		
Most favored (%)	87.8	89.9
Additionally allowed region (%)	10.7	9.0
Generously allowed region (%)	1.3	0.8
Disallowed region (%)	0.2	0.3

[a]RMSD to the mean structure for the ATP-lid (from the residue 98 to 136) calculated for the 20 structures with the lower CYANA target functions refined using CNS restrained molecular dynamics.
[b]Ramachandran statistics calculated with PROCHECK for both states of HSP90-NTD.

Supplementary Table 4: Pairwise RMSD comparison of MD and X-ray representative structures.

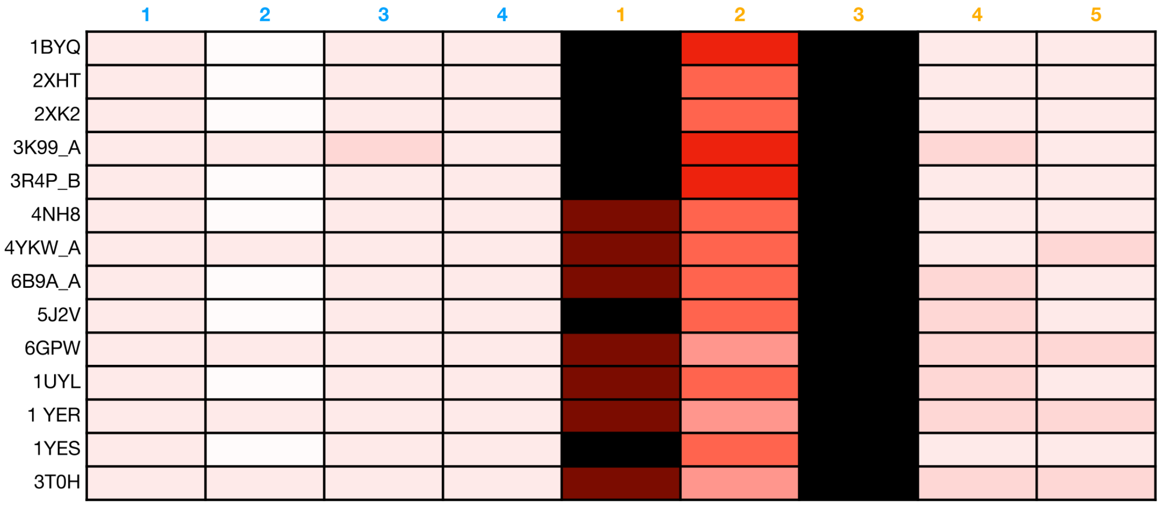

RMSD between 9 centroids representative of MD trajectories (4 in blue, 5 in orange obtained respectively for the ATP-lid open and closed states as presented on Fig. 4), the 8 centroids representing the PDB structures (Fig. 1) and the 6 available experimental apo structures (PDB codes: 5J2V, 6GPW, 1UYL, 1YER, 1YES, 3T0H) of WT-HSP90-NT. Structures were superimposed on the backbone of residues 11 to 97 and 137 to 220, and RMSD were calculated for Cα atoms from residues 98 to 136. The red, used to represent Cα RMSD, goes darker when the RMSD values increase. The 4 centroids emerging from the simulations starting from the open lid state have RMSD values between 1.9 and 5.5 Å with the experimental crystal structures. The closest pairs are between the centroid 2 and PDB structures: 2XHT and 2XK2. The 5 centroids representing the simulations starting from the closed lid state can be superimposed on backbone with RMSD values between 3.1 and 18.7 Å. The centroids 1, 2 and 3 represent conformation around the closed state and are very far from the crystal structures. The centroids 4 and 5 represent the conformation that have returned towards the ground state, and are closer to the HSP90α-NTD representative PDB structures.

Supplementary Table 5: Identification of characteristic distances of dimeric forms of HSP90α-NTD.

Close by pairs of methyl groups representative of a possible dimerization of HSP90-NTD with the ATP-lid closed state and intermolecular exchange of the two β_1 strands are shown column 1. Such pair of methyl groups correspond to inter-chain putative NOEs involving one methyl group from residue 13 to 41 and any other methyl group of the NTD for which the measured distance was ≤ 11 Å in the full-length dimer HSP90α in closed form (PDB: 7L7J) but > 11 Å for both the solution structure ensemble representative of the ground/open and excited/closed ATP-lid states (Fig. 3). In the second column, presence/absence of putative specific NOEs of HSP90α-NTD dimer have been investigated. Their presence/absence has been verified in the 3D $^{13}CH_3$-edited NOESY spectrum acquired using the methyl-labeled R46A-HSP90α-NTD sample.

Contacts (d_{HH} ≤ 11 Å) specific of dimeric full-length HSP90α	Present in NOESY spectra of R46A-HSP90-NTD ?
17 - 149	No
17 - 174	No
17 - 176	No
21 - 26	No
21 - 110	No
24 - 29	No
24 - 30	No
24 - 33	No
24 - 110	No
24 - 136	No
29 - 109	No
32 - 111	No
33 - 24	No
33 - 111	No
36 - 111	No
36 - 136	No

Supplementary Table 6: NMR-based structural calculations Supplementary the absence of helix-5 in ATP-lid closed state.

All CYANA structural calculations performed without angular restraints for helix-5, but including all experimental NOE-derived distance restraints have a target function below 5 $kJ.mol^{-1}$ and no violation of NMR experimental restraints. In contrast, when dihedral restraints are introduced to force the formation of helix-5, the target function increases above 6 $kJ.mol^{-1}$ and 3 to 4 violations of NMR structural restraints are observed.

Helices present in the structural calculation	Energy ($kJ.mol^{-1}$)	Distance restraint violation	Angular restraint violation
None	4.51 ± 0.04	⌀	⌀
α3	4.54 ± 0.05	⌀	⌀
α4	4.60 ± 0.04	⌀	⌀
α5	6.13 ± 0.06	2[a]	1[b]
α3α4	4.56 ± 0.02	⌀	⌀
α3α5	6.13 ± 0.08	2[a]	1[b]
α4α5	6.23 ± 0.09	2[a]	2[c]
α3α4α5	6.14 ± 0.07	2[a]	2[c]

[a] Violated distance restraints : THR65 HG2* - MET130 HE* and ILE131 HD1* - LEU143 HD2*
[b] Violated angular restraints: PHI SER211
[c] Violated angular restraints : PHI SER211 and CHI1 ILE214
⌀ : no violation

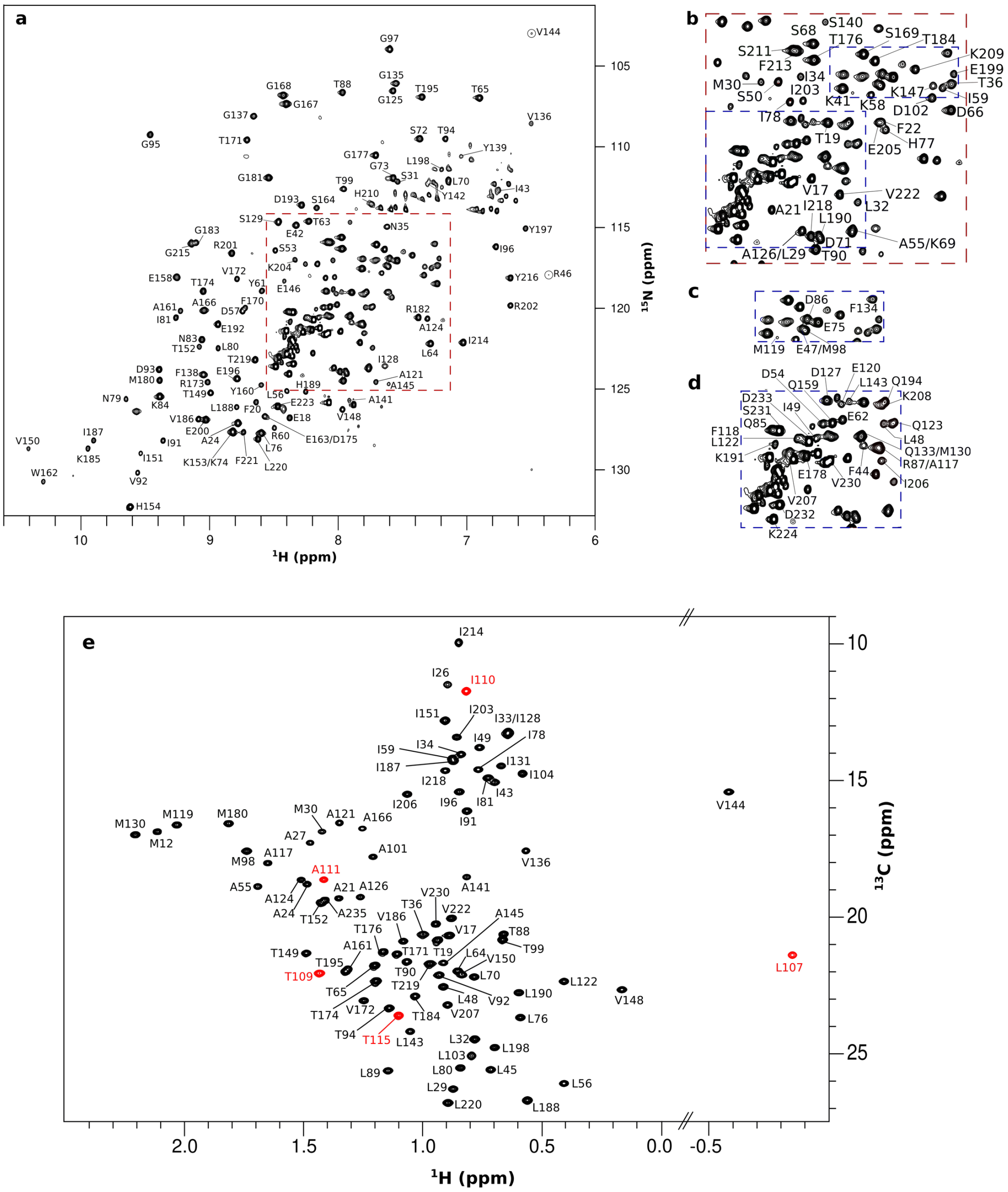

Supplementary Figure 1: Assigned 2D NMR spectra of HSP90α-NTD. **a)** ^{15}N-BEST-TROSY spectrum of HSP90α-NTD in its apo state, acquired at 293 K. Panels **b-d)** present zooms of 2D ^{15}N-BEST-TROSY spectrum. Each assigned signal (a, b, c, d) is annotated with the corresponding residue number[47-49]. Panel **e)** displays the fully assigned 2D ^{1}H-^{13}C SOFAST methyl TROSY spectrum of HSP90α-NTD apo, recorded at 298 K. Each signal is annotated with the corresponding residue number[49]. In red are displayed residues belonging to the stretch [105-116] invisible on ^{15}N-BEST-TROSY due to conformational exchange in the µs-ms timescale.

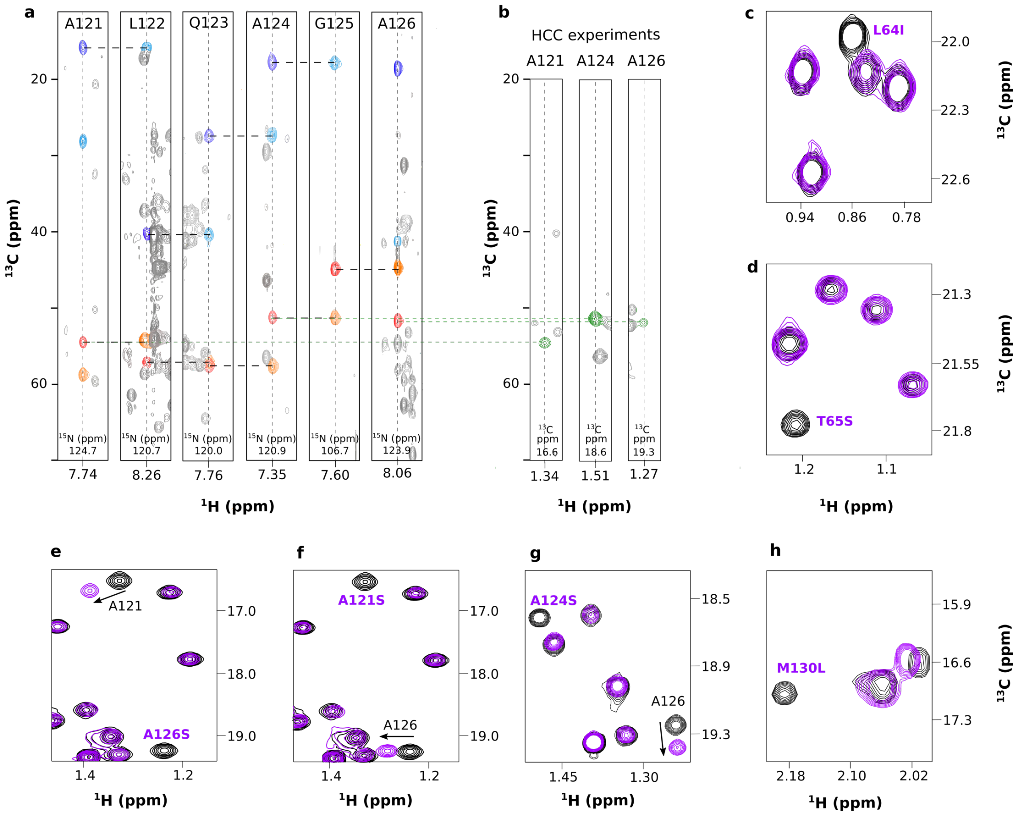

Supplementary Figure 2: HSP90α-NTD assignment. a) Examples of 2D-extracts from 3D HNCA, HN(CO)CA, HN(CA)CB, HN(COCA)CB showing backbone correlations from A121 to A126. Spectra were acquired at 298 K on a NMR spectrometer operating at a ^{1}H frequency of 600 MHz using a U-[^{2}H, ^{13}C, ^{15}N]-HSP9α-NTD sample. $C\alpha_{(i)}$, $C\alpha_{(i-1)}$, $C\beta_{(i)}$, $C\beta_{(i-1)}$, are colored in red, orange, dark blue and light blue, respectively. **b)** Examples of 2D-extracts from 'out and back' HCC experiment to transfer assignment from backbone atoms to the methyl groups**.** **c-h)** Cross-validation of assignment of methyl groups using mutagenesis. Examples of 2D ^{1}H-^{13}C SOFAST methyl TROSY spectra of 6 HSP90α-NTD mutants. Each mutant spectrum extract (purple) is superimposed with the wild type protein (black). A total of 56 single point amino acid mutations were generated by GeneCust and for each of these samples a single type of methyl group was labeled by addition of the corresponding NMR-Bio kit (SLAM-A^{β}, SLAM-M^{ε}, SLAM-$I^{\delta 1}$, SLAM-T^{γ}, SLAM-V^{proS} or DLAM-LV^{proS}) in M9/$^{2}H_2O$ media. The following mutants were produced and analyzed by NMR: M12L, M30L M119L, M130L, M180L, A21S, A24S, A27S, A55S, A101S, A111S, A117S, A121S, A124S, A126S, A161S, A166S, A235S, T19S, T36S, T65S, T99S, T109S, T115S, T152S, T171S, T174S, T184S, T195S, T219S, I26V, I33V, I34V, I81V, I104V, I110V, I128V, I131V, I214V, V17I, V136I, V150I, V172I, V186I, V222I, V230I, L29I, L32I, L45I, L48I, L56I, L64I, L70I, L107A, L122I, L143I.

A

B

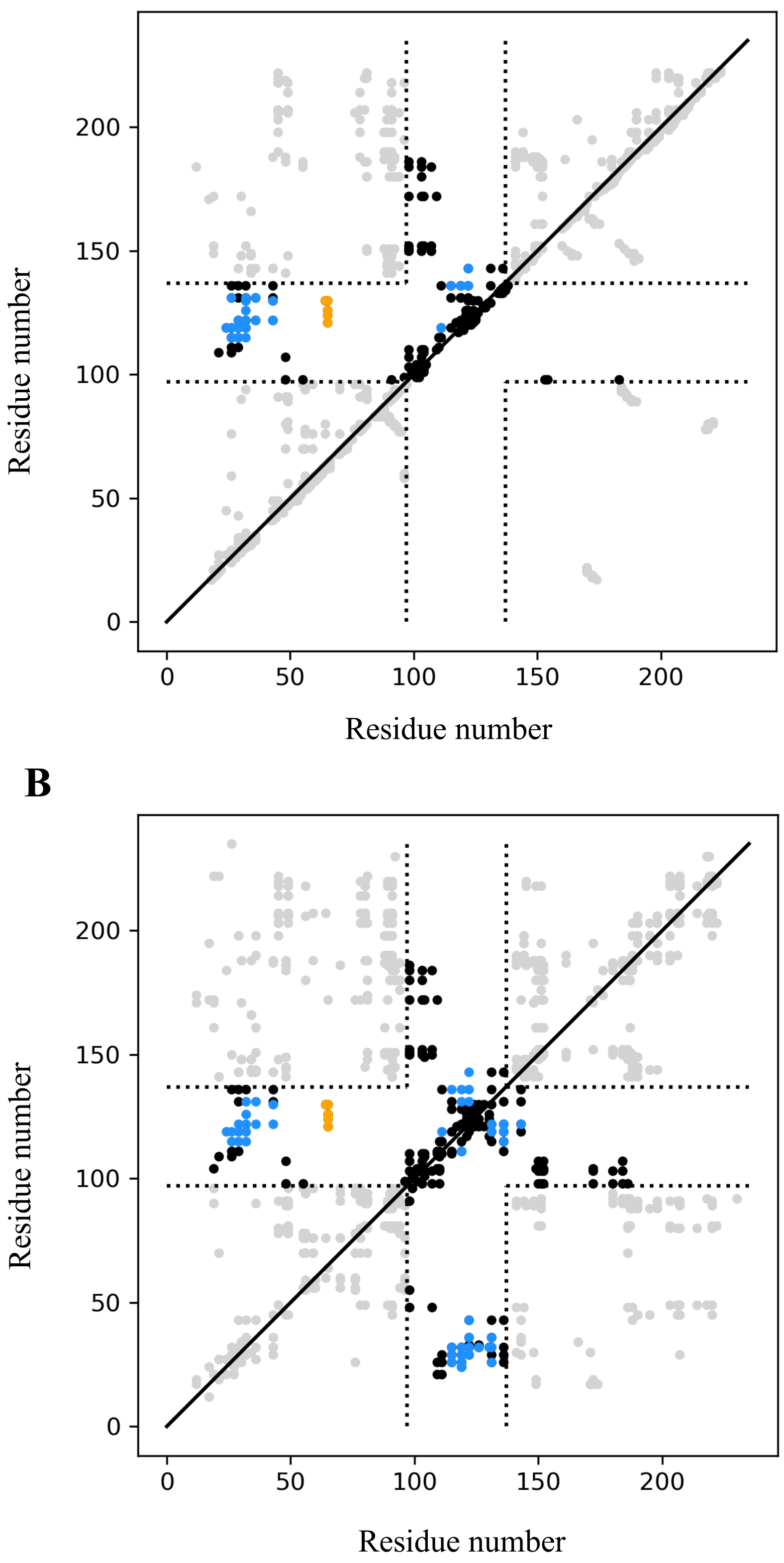

Supplementary Figure 3: Summary of NOEs distance restraints. A) Experimentally detected CH_3-CH_3 (top left) and H_N-H_N (bottom right) NOEs in WT-HSP90α-NTD. **B)** Experimentally detected CH_3-CH_3 NOEs for R46A- (top left) and R60A- (bottom right) HSP90α-NTD. Black points represent NOEs of the ATP-lid present in both states. Blue/orange points represent NOEs specific of ATP-lid in open/closed states, respectively (Supplementary Table 2). Dot lines indicate the positions of residues 98 and 136.

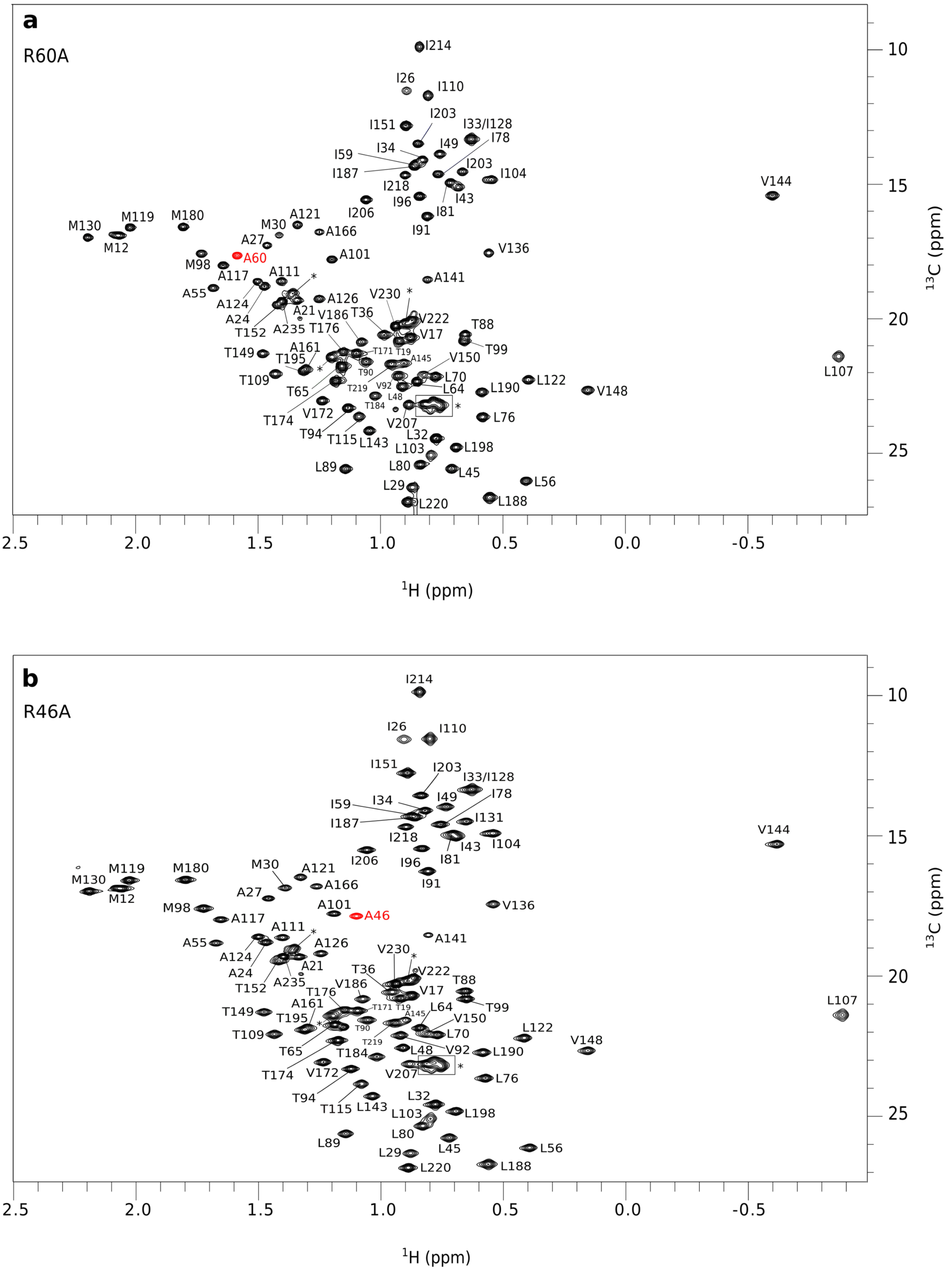

Supplementary Figure 4: Assigned 2D ^{1}H-^{13}C SOFAST methyl TROSY of HSP90α-NTD mutants. a) R60A- and **(b)** R46A-HSP90α-NTD mutants, recorded at 293 K and 288 K, respectively. Each signal is annotated with the corresponding residue number. Peaks annotated with the symbol * are peaks belonging to the uncleaved N-terminal tag. Extra alanine methyl is colored in red.

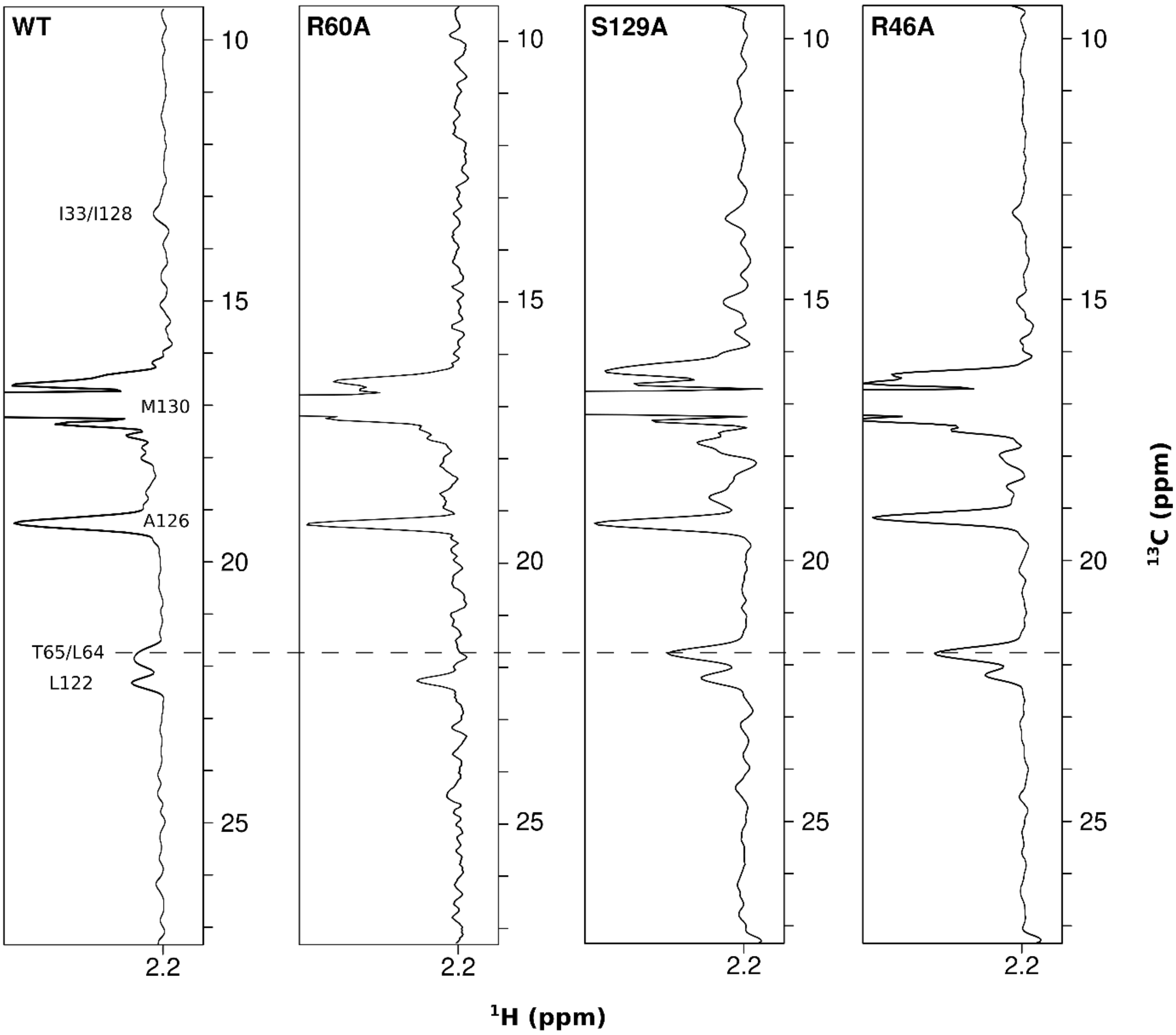

Supplementary Figure 5: Intensity variation of characteristic NOEs. 1D traces extracted from 3D CCH HMQC-NOESY-HMQC experiments showing M130 as diagonal peak and associated NOEs cross peaks for the wild type protein HSP90α-NTD and three mutants: R60A, S129A and R46A. 3D ^{13}C-edited NOESY spectra were acquired during *ca.* 2 days each using high-field NMR spectrometers operating at 950 or 850 MHz and at a temperature of 25°C (WT), 20°C (R60A) or 15°C (R46A), taking into account the lower stability of mutants. NOE mixing time were optimized to delays ranging from 0.3 (15°C) to 0.4 s (25°C), corresponding to the estimated maximum intensities of NOEs buildups for each experimental condition.

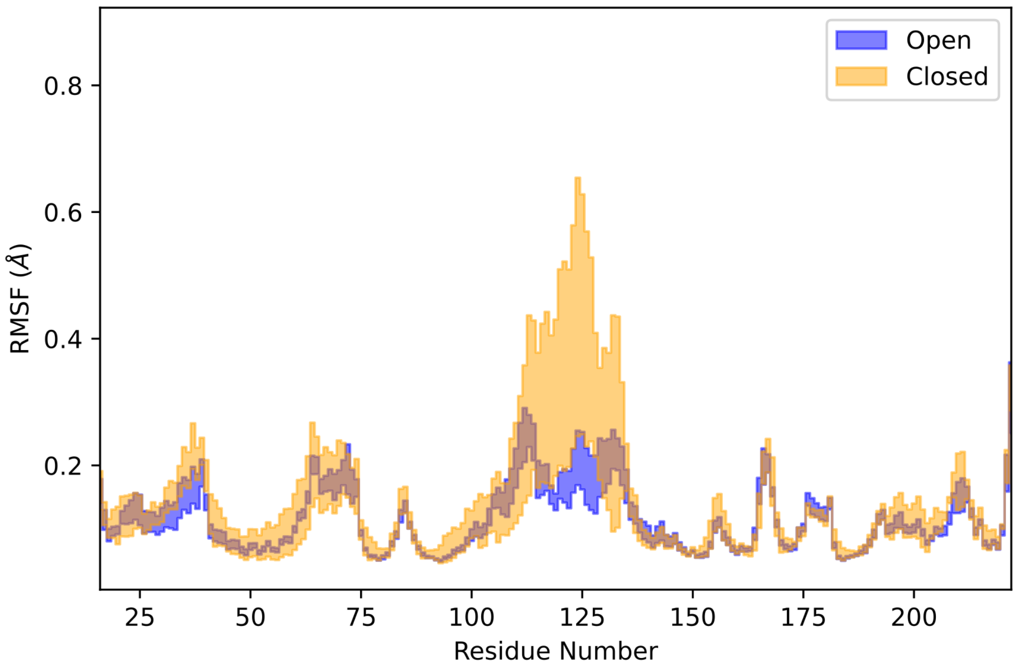

Supplementary Figure 6: Root mean square fluctuation analysis of MD trajectories. RMSF values are first calculated residue-wise for each of the 1 microsecond simulations that remain stable. Then, the average and standard error are calculated for each residue, grouping on the one hand the 20 stable simulations starting from the open lid-state (blue), on the other hand the 4 stable ones starting from the closed lid-state (yellow). For each residue number, the filling is done around the average value with a thickness of two times the standard errors of the mean.

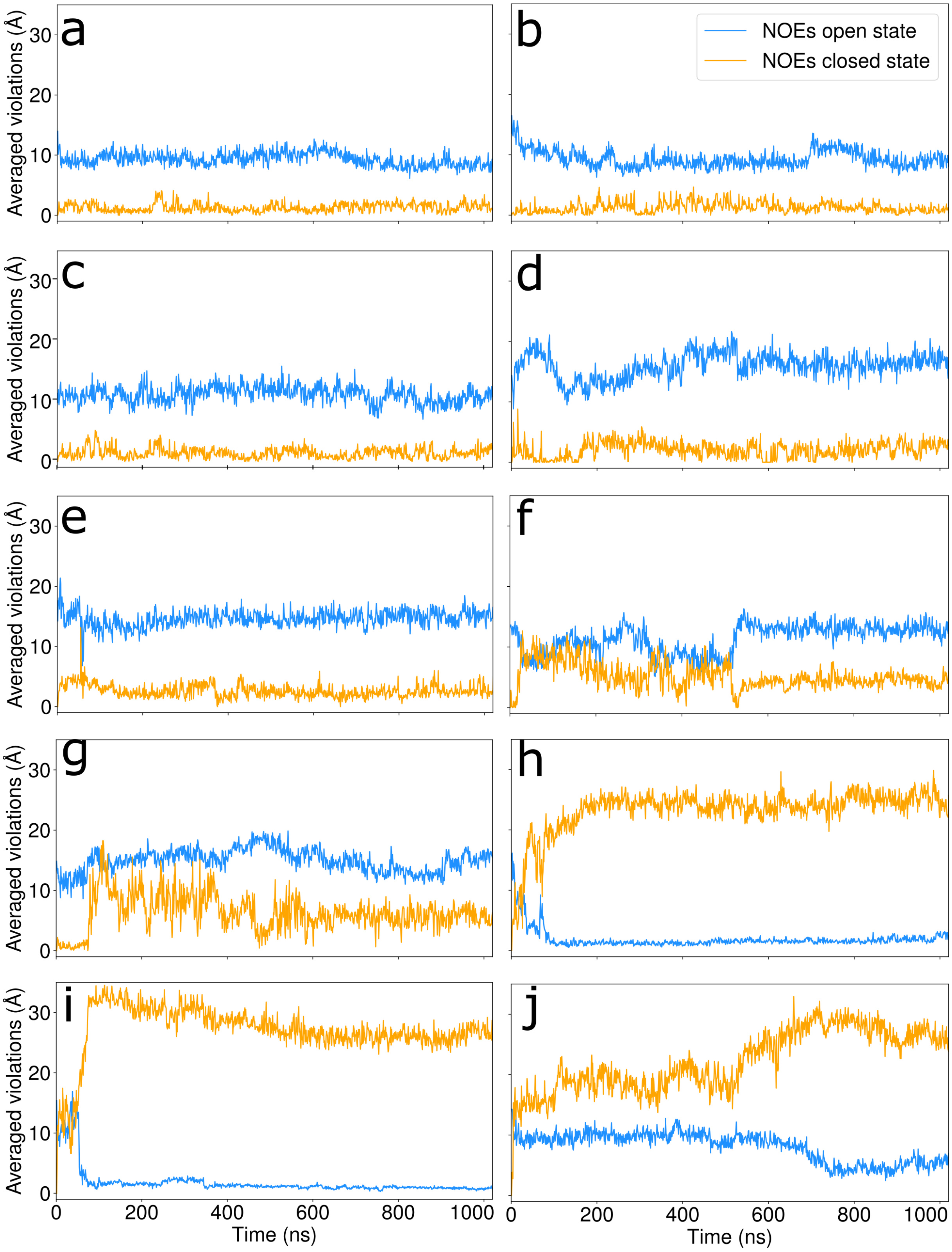

Supplementary Figure 7a: Time evolution of the violations of the NOE-derived distance restraints specific of either the open or the closed conformation (orange and blue respectively), for the 20 non-restrained molecular dynamics simulations starting from ATP-lid closed state conformers. The violations are defined in section 1.2.2 of Methods, and the simulations were classified according to the violations. **a-c**: relatively stable trajectories, compatible with closed-state-NOE distances during several hundreds of nanoseconds. **d-g**: relatively stable trajectories, remaining nearby the closed state during several hundreds of nanoseconds. **h-j**: unstable trajectories with transition toward a conformation close to the open-state.

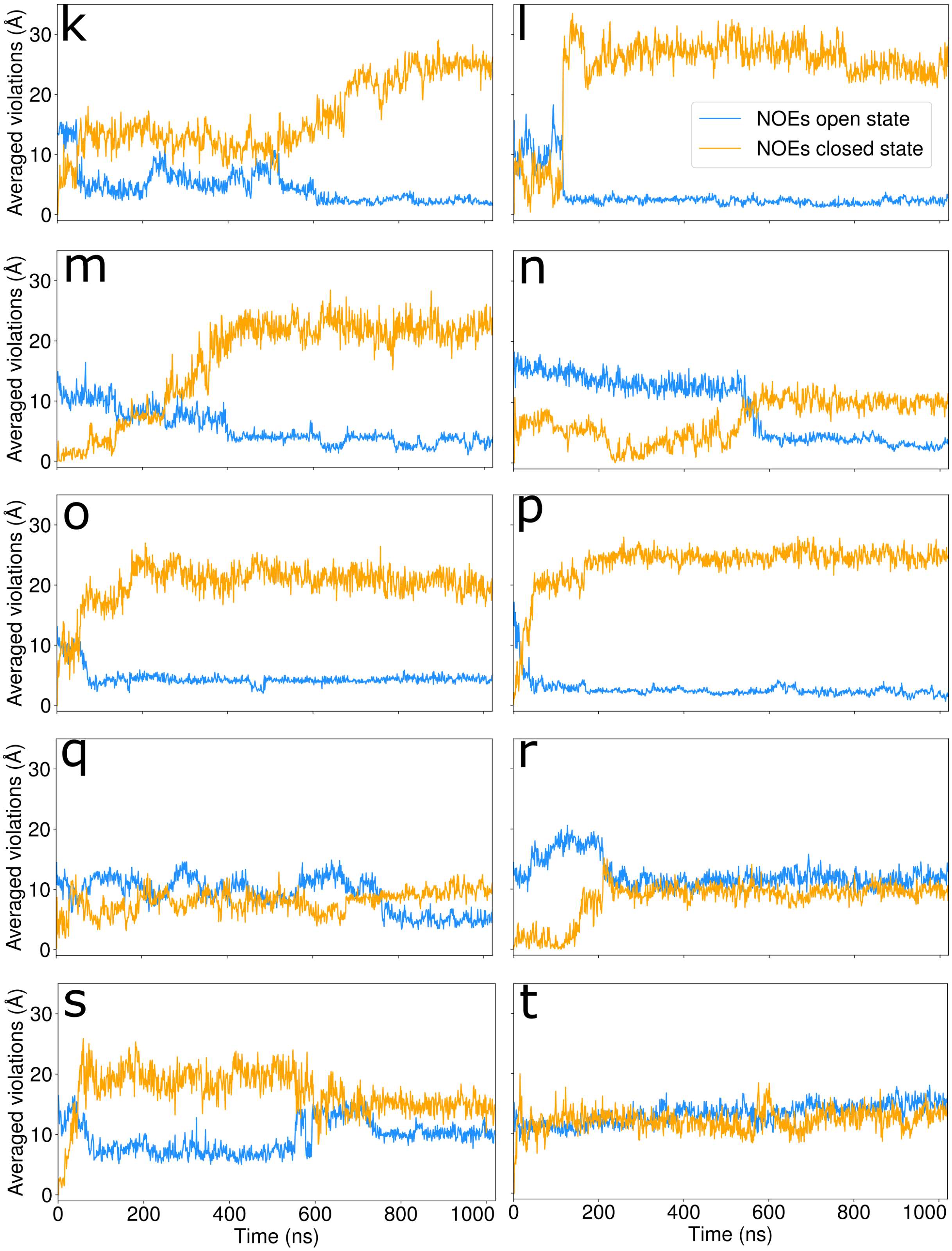

Supplementary Figure 7b: same as Supplementary Figure S7a. k-p: unstable trajectories with transition toward a conformation close to the open-state. **q-t**: unstable trajectories with transition toward a conformation neither closes nor open-state.

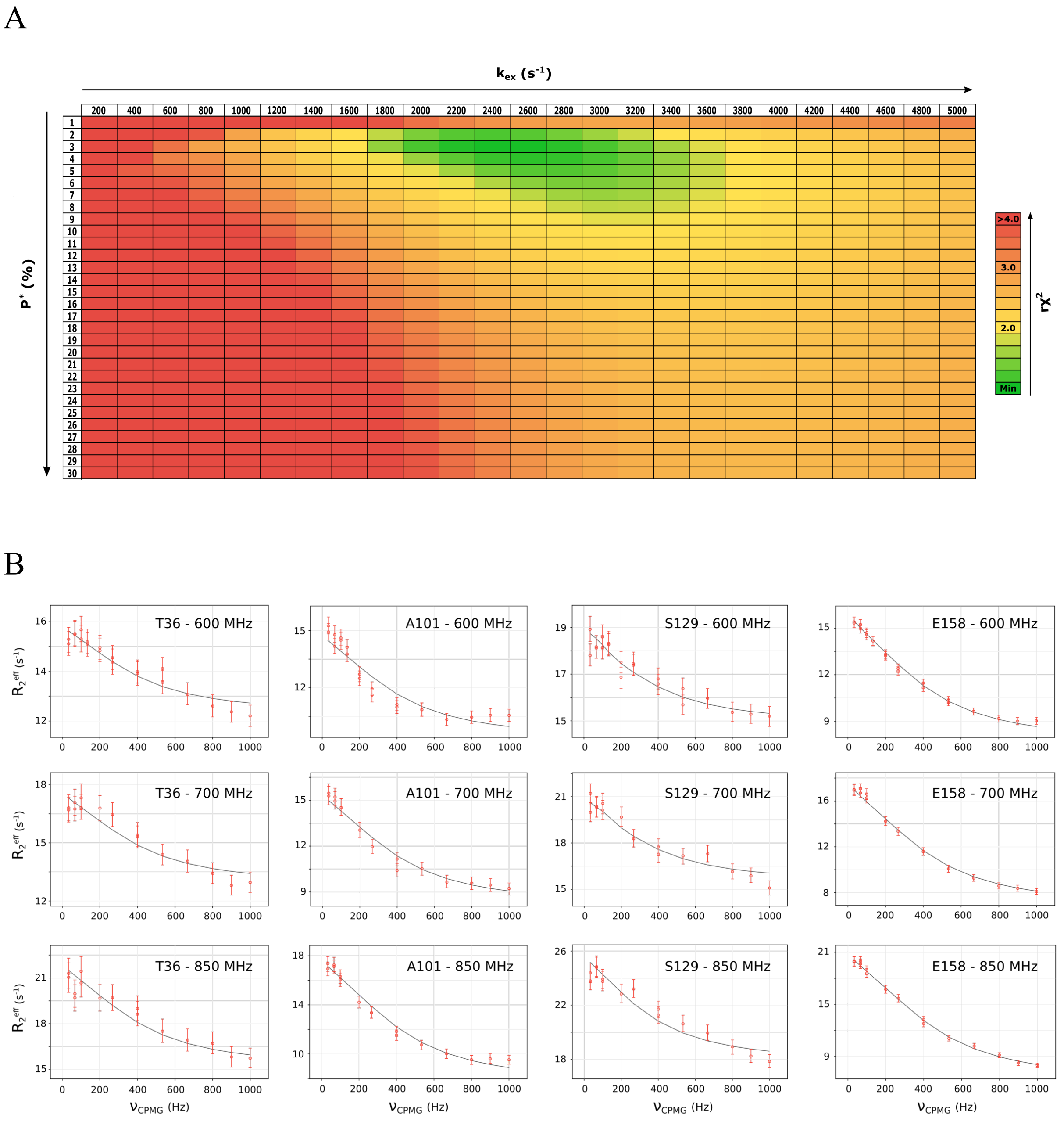

Supplementary Figure 8: Analysis of HSP90-NTD dynamics using relaxation dispersion experiments. A) Grid search table displaying the reduced χ^2 of the fit obtained from ^{13}C-relaxation data acquired at 293 K, when exchange rate and population of the excited state were fixed. Going from green to red the reduced χ^2 values increase. **B)** Global fitting of ^{15}N relaxation dispersion data of HSP90α-NTD-WT obtained at 293 K at three different magnetic field strengths. Graphs representing experimental R_2^{eff} as a function of the CPMG frequency (ν_{CPMG}) obtained for four residues: T36, A101, S129 and E158 out of 14 residues fitted globally (global fit represented by the black curves). Errors for $R_{2,eff}$ rate values were estimated from twice the noise measured in the spectra. However, when errors were less than 2% of the $R_{2,eff}$ rate value, an error of 2% was assumed. The conformational exchange rate and population of the excited state obtained with a global fit of ^{15}N relaxation dispersion experiments are 2435 +/- 89 s^{-1} and 2.5 +/- 0.4 %, respectively.

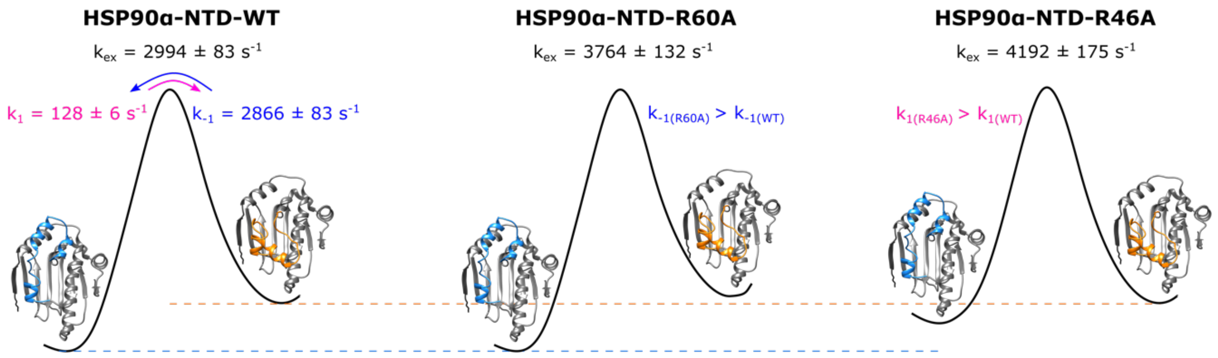

Supplementary Figure 9: **Schematic diagrams of the energy landscapes for the exchange between the ATP-lid open (ground) and closed (excited) states of WT-, R60A-, R46A-HSP90α-NTD**. Kinetic parameters of the exchange, extracted at 288 K, are displayed both for the WT protein and the protein mutants. Assuming, that the mutations would not influence the energy of the transition state and would destabilize only the state for which the segment [98-136] is in close proximity to the mutation, we could deduce that the R46A (R60A) mutation will induce an increase of k_1 (respectively k_{-1}) and therefore of the global exchange constant $k_{ex} = k_1 + k_{-1}$ as experimentally observed.

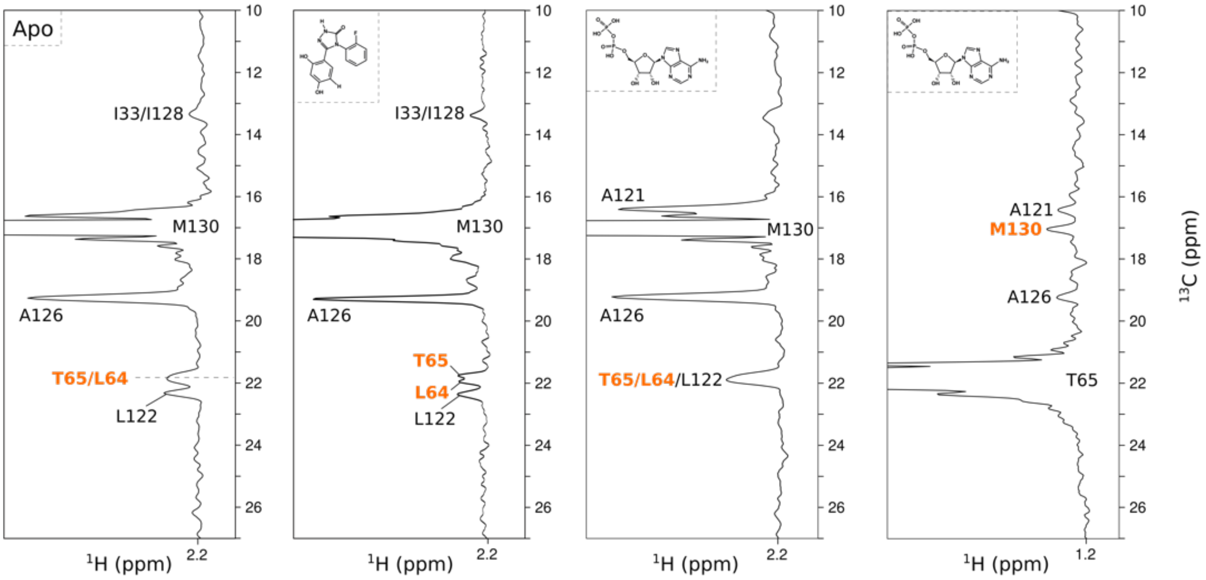

Supplementary Figure 10: Intensity variation of closed-state-characteristic NOEs in presence of ligands. 1D traces extracted from 3D CCH HMQC-NOESY-HMQC spectra showing M130 as a diagonal peak and its associated NOE cross peaks for apo-HSP90-NTD, HSP90 in complex with a resorcinol derivative: 5-(2,4-dihydroxy-phenyl)-4-(2-fluoro-phenyl)-2,4-dihydro-[1,2,4]triazol-3-one and HSP90-NTD bound to ADP. Additionally, for HSP90-NTD in complex with ADP a 1D trace showing T65 as a diagonal peak and its associated NOE cross peaks is displayed. NOE cross peaks annotated in orange are characteristic of the ATP-lid in closed state. 3D ^{13}C-edited NOESY spectra were acquired during *ca.* 2-3 days each using high-field NMR spectrometers operating at 950 or 850 MHz and at a temperature of 25°C.

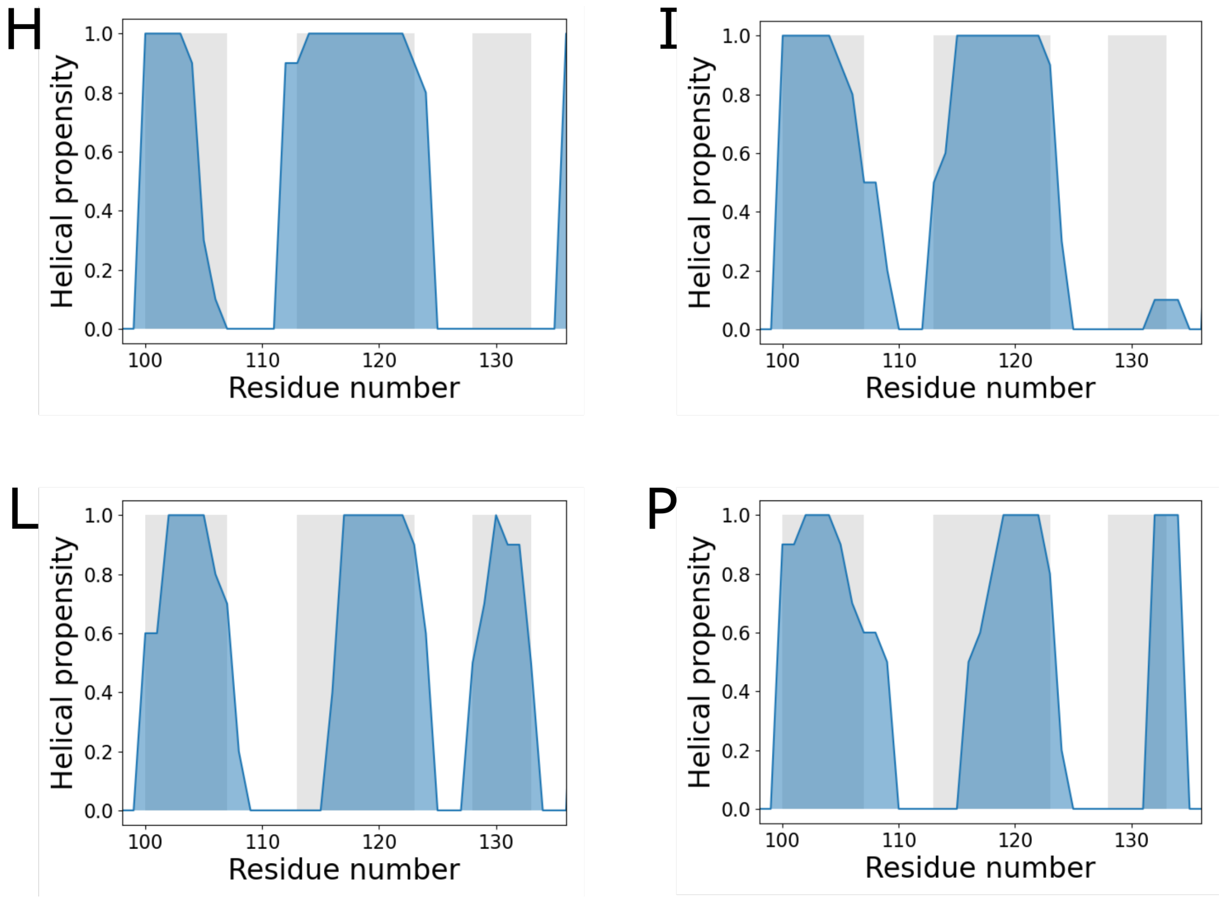

Supplementary Figure 11: Probability of helical structures along the sequence of the ATP lid, in 4 MD simulations depicting a transition of the ATP-lid from a closed to an open state. The helical propensities are shown in blue. For comparison, the helical regions of the ground state are represented in gray (helices 3, 4 and 5). Each panel is annotated with the letter corresponding to panel of Supplementary Fig. 7, presenting the 20 different MD simulations starting from closed-state conformers. These four trajectories were selected on the basis of their violations of the specific NOE of the open state, which were lower than *ca*. 3.5 Å. On average over these four selected simulations, the helical propensity of the helix-5 (residues [128-134]) is 28 ± 18%. In comparison, on average over the 20 simulations on the ground state, the helical propensity of the helix-5 is much higher: 63 ± 5% on the last 120 ns. Therefore, the formation of the helix-5 is not systematically observed after a swap of the ATP-lid from the closed to the open state.

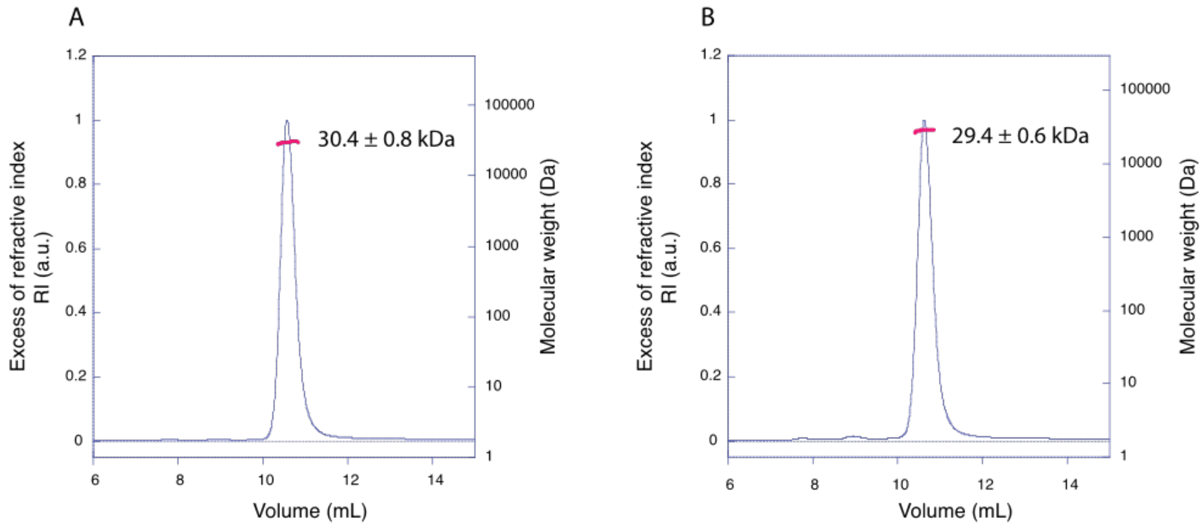

Supplementary Figure 12: SEC-MALS analysis of uncleaved R46A-HSP90α-NTD (A) and WT-HSP90α-NTD (B) using gel filtration column Superdex 75 PG (GE Healthcare) equilibrated with NMR buffer.
