# Supplementary material for: Visualizing the transiently populated closed-state of human HSP90 ATP binding domain
Source: Nat Commun. 2022 Dec 9;13:7601. doi: 10.1038/s41467-022-35399-8 (PMC9734131; doi:10.1038/s41467-022-35399-8)
Supplement: Supplementary file 3 — Reporting Summary [file 41467_2022_35399_MOESM3_ESM.pdf]

# nature portfolio

Corresponding author(s): J. Boisbouvier & C. Loison

Last updated by author(s): 11/25/2022



# Reporting Summary

Nature Portfolio wishes to improve the reproducibility of the work that we publish. This form provides structure for consistency and transparency in reporting. For further information on Nature Portfolio policies, see our Editorial Policies and the Editorial Policy Checklist.

## Statistics

For all statistical analyses, confirm that the following items are present in the figure legend, table legend, main text, or Methods section.

| n/a | Confirmed | |
|---|---|---|
| ☐ | ☒ | The exact sample size (*n*) for each experimental group/condition, given as a discrete number and unit of measurement |
| ☐ | ☒ | A statement on whether measurements were taken from distinct samples or whether the same sample was measured repeatedly |
| ☒ | ☐ | The statistical test(s) used AND whether they are one- or two-sided<br>*Only common tests should be described solely by name; describe more complex techniques in the Methods section.* |
| ☒ | ☐ | A description of all covariates tested |
| ☒ | ☐ | A description of any assumptions or corrections, such as tests of normality and adjustment for multiple comparisons |
| ☐ | ☒ | A full description of the statistical parameters including central tendency (e.g. means) or other basic estimates (e.g. regression coefficient) AND variation (e.g. standard deviation) or associated estimates of uncertainty (e.g. confidence intervals) |
| ☒ | ☐ | For null hypothesis testing, the test statistic (e.g. *F*, *t*, *r*) with confidence intervals, effect sizes, degrees of freedom and *P* value noted<br>*Give P values as exact values whenever suitable.* |
| ☐ | ☒ | For Bayesian analysis, information on the choice of priors and Markov chain Monte Carlo settings |
| ☒ | ☐ | For hierarchical and complex designs, identification of the appropriate level for tests and full reporting of outcomes |
| ☒ | ☐ | Estimates of effect sizes (e.g. Cohen's *d*, Pearson's *r*), indicating how they were calculated |

*Our web collection on statistics for biologists contains articles on many of the points above.*

## Software and code

Policy information about availability of computer code

| | |
|---|---|
| Data collection | For the collection of data, the following softwares were used: topSpin 3.5, NMRlib2.0 and Gromacs2020.4,<br>All these softwares are described in published literature and available to the scientific community. |
| Data analysis | For the analysis of experimental data, the following softwares were used: nmrPipe9.4; ccpnmr analysis 2.4; CYANA 3.98.13, CNS, ChemEx2018.9.4,Talos +, Gromacs2020.4,, H++ 3.2 (web server), MaxCluster, MDanalysis2.0.0 and TTClust4.10.1. All these softwares are described in published literature and available to the scientific community. |

For manuscripts utilizing custom algorithms or software that are central to the research but not yet described in published literature, software must be made available to editors and reviewers. We strongly encourage code deposition in a community repository (e.g. GitHub). See the Nature Portfolio guidelines for submitting code & software for further information.

## Data

Policy information about availability of data

All manuscripts must include a data availability statement. This statement should provide the following information, where applicable:

- Accession codes, unique identifiers, or web links for publicly available datasets
- A description of any restrictions on data availability
- For clinical datasets or third party data, please ensure that the statement adheres to our policy

The NMR structural and dynamics data together with corresponding NMR experiments used in this study are publicly available in the Biological Magnetic Resonance

Data Bank under the code bmrbig44[https://bmrbig.org/released/bmrbig44] and Protein Data Bank under the codes 8B7I[http://doi.org/10.2210/pdb8B7I/pdb] and 8B7J[http://doi.org/10.2210/pdb8B7J/pdb] .
Several structures previously deposited in the PDB were used in this article, they are publicly available in Protein Data Bank under the codes: 1YES[http://doi.org/10.2210/pdb1YES/pdb], 2XHT[http://doi.org/10.2210/pdb2XHT/pdb], 2XK2[http://doi.org/10.2210/pdb2XK2/pdb], 4NH8[http://doi.org/10.2210/pdb4NH8/pdb], 3K99_A[http://doi.org/10.2210/pdb3K99/pdb], 3R4P_B[http://doi.org/10.2210/pdb3R4P/pdb], 4YKW_A[http://doi.org/10.2210/pdb4YKW/pdb], 6B9A_A [http://doi.org/10.2210/pdb6B91/pdb], 1BYQ[http://doi.org/10.2210/pdb1BYQ/pdb], 7L7J[http://doi.org/10.2210/pdb7L7J/pdb].
The Molecular Dynamics and analysis have been deposited on Zenodo repository under the code doi: 10.5281/zenodo.6606744.

## Human research participants

Policy information about studies involving human research participants and Sex and Gender in Research.

| | |
|---|---|
| Reporting on sex and gender | Not Applicable |
| Population characteristics | Not Applicable |
| Recruitment | Not Applicable |
| Ethics oversight | Not Applicable |

Note that full information on the approval of the study protocol must also be provided in the manuscript.

# Field-specific reporting

Please select the one below that is the best fit for your research. If you are not sure, read the appropriate sections before making your selection.

[x] Life sciences [ ] Behavioural & social sciences [ ] Ecological, evolutionary & environmental sciences

For a reference copy of the document with all sections, see nature.com/documents/nr-reporting-summary-flat.pdf

# Life sciences study design

All studies must disclose on these points even when the disclosure is negative.

| | |
|---|---|
| Sample size | The sample size for NMR experiments were chosen according to the NMR tubes (i.e. 200 microL for a 4 mm shigemi tube) and maximal solubility of the protein (0.5 mM). Individual samples were measured in experiments where comparative rather than statistical analyses were to be performed ( NMR as well as MD simulations). |
| Data exclusions | No data were exluded from the analysis |
| Replication | All 2D NMR experiments were acquired at least 3 times with similar results. The 3D 13C-edited NOESY experiments of apo WT-HSP90-NTD were acquired twice using two different samples with similar results. |
| Randomization | Not Applicable. Randomization is used in animal studies or clinical trials and does not apply to NMR experiments or MD simulations. |
| Blinding | Not applicable. This study does not include any animal studies, clinical trials or research. |

# Reporting for specific materials, systems and methods

We require information from authors about some types of materials, experimental systems and methods used in many studies. Here, indicate whether each material, system or method listed is relevant to your study. If you are not sure if a list item applies to your research, read the appropriate section before selecting a response.

## Materials & experimental systems

| n/a | Involved in the study | |
|---|---|---|
| [x] | [ ] | Antibodies |
| [x] | [ ] | Eukaryotic cell lines |
| [x] | [ ] | Palaeontology and archaeology |
| [x] | [ ] | Animals and other organisms |
| [x] | [ ] | Clinical data |
| [x] | [ ] | Dual use research of concern |

## Methods

| n/a | Involved in the study | |
|---|---|---|
| [x] | [ ] | ChIP-seq |
| [x] | [ ] | Flow cytometry |
| [x] | [ ] | MRI-based neuroimaging |
